# *Tenebrio molitor*: possible source of polystyrene-degrading bacteria

**DOI:** 10.1186/s12896-021-00733-3

**Published:** 2022-01-04

**Authors:** Oleen Machona, Farisai Chidzwondo, Rumbidzai Mangoyi

**Affiliations:** Department of Biotechnology and Biochemistry, University of Zimbabwe, Harare, Zimbabwe

**Keywords:** *Tenebrio molitor*, Polystyrene, Mealworms, Biodegradation, *Klebsiella oxytoca* ATCC 13182, *Klebsiella oxytoca* NBRC 102593, *Klebsiella oxytoca* JCM 1665

## Abstract

**Background:**

The excessive use of polystyrene as a packaging material has resulted in a rise in environmental pollution. Polystyrene waste has continually increased water pollution, soil pollution and the closing of landfill sites since it is durable and resistant to biodegradation. Therefore, the challenge in polystyrene disposal has caused researchers to look for urgent innovative and eco-friendly solutions for plastic degradation. The current study focuses on the isolation and identification of bacteria produced by the larvae of beetle *Tenebrio molitor* (yellow mealworms), that enable them to survive when fed with polystyrene foam as their sole carbon diet.

**Materials and methods:**

The biodegradation of polystyrene by *Tenebrio molitor* was investigated by breeding and rearing the mealworms in the presence and absence of polystyrene. A comparison was made between those fed with a normal diet and those fed on polystyrene. The mealworms which were fed with polystyrene were then dissected and the guts were collected to isolate and identify the bacteria in their guts. The viability and metabolic activity of the isolates were investigated. The polymerase chain reaction (PCR) followed by sequencing was used for molecular identification of the isolates. The PCR products were directly sequenced using Sanger’s method and the phylogenetic tree and molecular evolutionary analyses were constructed using MEGAX software with the Neighbour Joining algorithm. The evolutionary distances were computed using the Maximum Composite Likelihood method.

**Results:**

The decrease in mass of the polystyrene as feedstock confirmed that the mealworms were depending on polystyrene as their sole carbon diet. The frass egested by mealworms also confirmed the biodegradation of polystyrene as it contained very tiny residues of polystyrene. Three isolates were obtained from the mealworms guts, and all were found to be gram-negative. The sequencing results showed that the isolates were *Klebsiella oxytoca* ATCC 13182, *Klebsiella oxytoca* NBRC 102593 and *Klebsiella oxytoca* JCM 1665.

**Conclusion:**

*Klebsiella oxytoca* ATCC 13182, *Klebsiella oxytoca* NBRC 102593 and *Klebsiella oxytoca* JCM 1665 maybe some of the bacteria responsible for polystyrene biodegradation.

**Supplementary Information:**

The online version contains supplementary material available at 10.1186/s12896-021-00733-3.

## Background

The excessive use of durable and degradation-resistant synthetic polymers such as polystyrene as a packaging material has resulted in a rise in environmental pollution [1]. Polystyrene, whose trade name is Styrofoam, stands to be the most widely used plastic with the scale of its production reaching quite a few million tons every year [2]. Polystyrene slowly fragments into nano-plastics which are harmful and have got detrimental consequences on ecosystems, biota, and the environment as well on the economy and human health [3]. Residues of plastics have been found in the stomach contents of many organisms such as earthworms, birds, turtles, dolphins and whales [4]. Fish and other marine organisms may continuously ingest small amounts of polystyrene and pose a health risk when consumed by people. The long term exposure to small quantities of styrene might cause neurotoxic effects which include fatigue, nervousness, sleeping difficulties, hematological effects which involve low platelet and haemoglobin values, cytogenetic effects which involve chromosomal and lymphatic abnormalities, and carcinogenic effects [5].

However, since the 1950s, researchers have observed that yellow mealworms, which are known as the larvae of *Tenebrio molitor* damaged plastic packaging materials [6]. The consumption of polystyrene foams by mealworms was later on reported by students that were competing in high school science fairs in 2003, whereby mealworms were raised by feeding them with polystyrene foam [7]. Biodegradation of polystyrene by mealworms has also been confirmed by academic researchers from different countries who concluded that yellow mealworms can survive when fed with polystyrene foam as their sole carbon diet [8]. Thus, the current study focuses on the isolation and identification of bacteria produced by the larvae of beetle *Tenebrio molitor*, which enable them to survive when fed with polystyrene foam. The gut of the larvae of *Tenebrio molitor* may contain microorganisms that degrade polystyrene, hence, yellow mealworms are economically among the most important species that could be used for biodegradation of polystyrene [8, 9].

## Results

### Characterization of polystyrene as feedstock, mealworms and mealworm frass

**Fig. 1:**
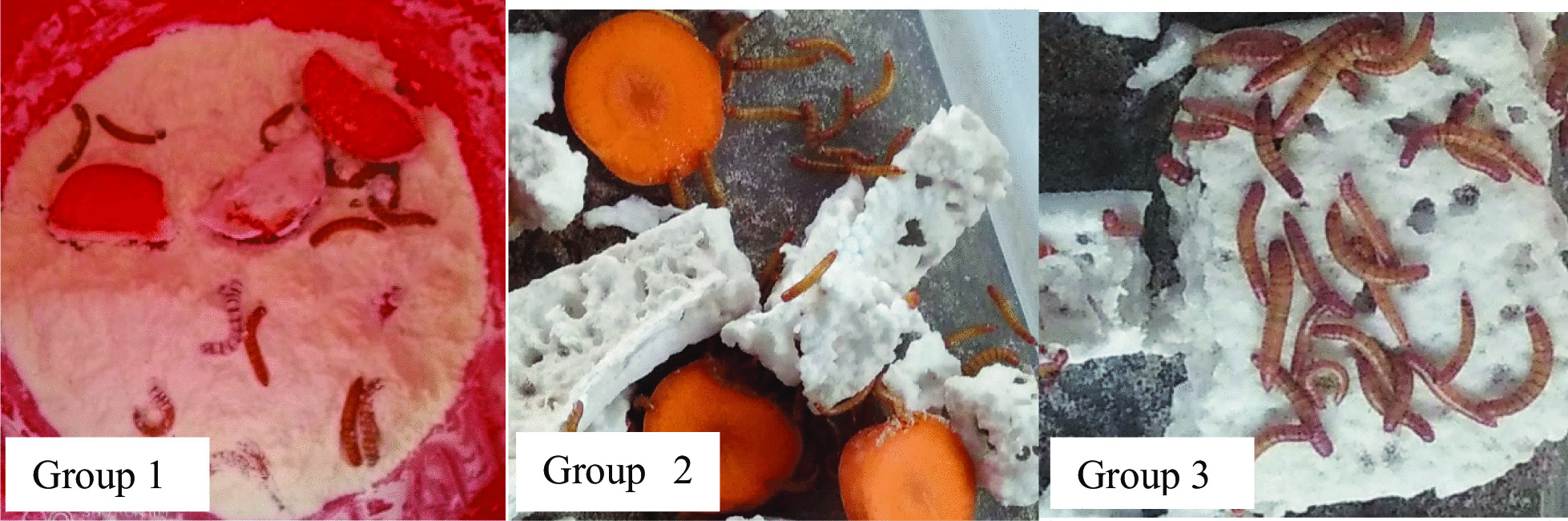
The larvae of *T. molitor* (mealworms) reared in the presence of corn flour (Group 1), polystyrene and carrots (Group 2) and polystyrene only (Group 3)

Biodegradation and depolymerization of ingested polystyrene were assessed by visual observation of both the polystyrene feedstock and frass. The hollows observed on polystyrene in groups 2 and 3 show that the mealworms were feeding themselves with polystyrene, hence, the mealworms were able to survive by virtue of their reserves within the 7 day test period, when they were fed with polystyrene foam. Consumption of the plastic polymer was aided by the gut microbiota as shown in Fig. 1.

**Fig. 2:**
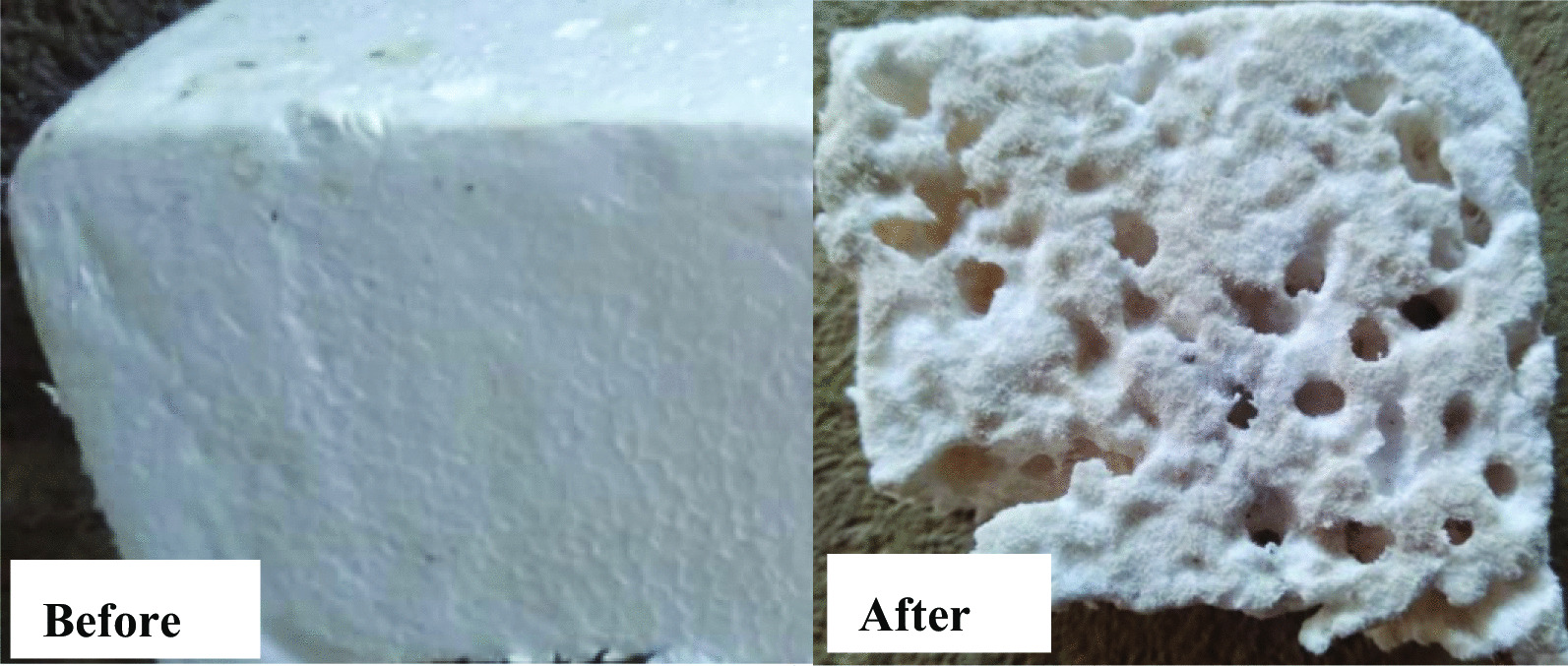
The changes observed on the polystyrene feedstock before and after it was fed to *Tenebrio molitor* mealworms for two months

Overall, there were visual changes that occurred to the polystyrene feedstocks after they were consumed by the mealworms which involved roughening of the surface, creation of holes and cracks. The general changes are shown in Fig. 2.

**Fig. 3:**
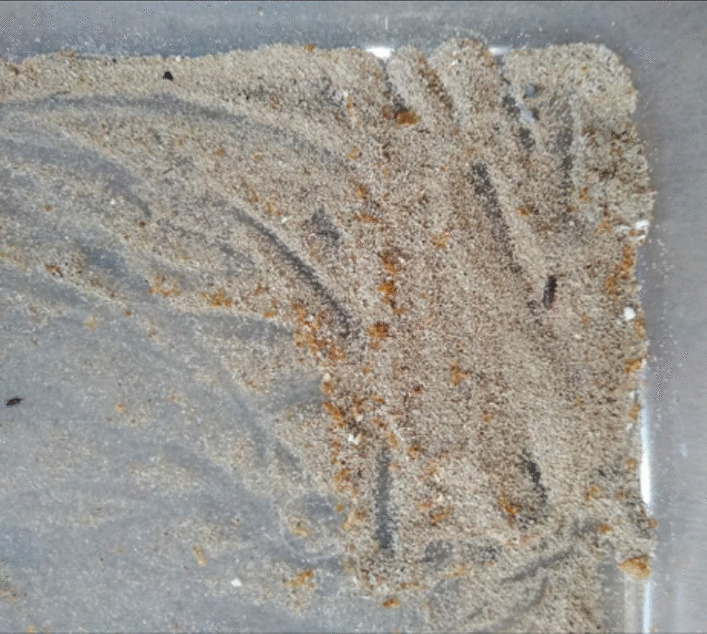
Frass excreted by the *Tenebrio molitor* mealworms after being fed with polystyrene

Figure 3 shows the frass egested by *Tenebrio molitor* mealworms. Further work needs to be done to investigate the constituents of the frass.

**Fig. 4:**
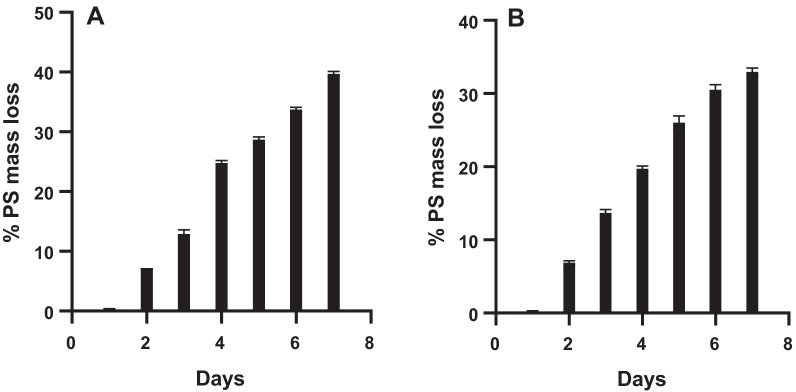
Mass loss in polystyrene. **A** is for group 2 that was fed with polystyrene and carrots whereas **B** is for group 3 that was fed with polystyrene only (PS = polystyrene). All values are mean ± SD for N = 2

The decrease in mass of the polystyrene as feedstock also confirmed that the mealworms were consuming polystyrene (aided by gut microbiota). Thus, the mass loss was recorded on daily basis and results are shown in Fig. 4. The results show the decrease in mass of the polystyrene from the initial mass of 1.5 g over seven days in groups 2 and 3. There was a 40% decrease in the mass of polystyrene for group 2 mealworms and a 33.33% decrease for group 3.

**Fig. 5:**
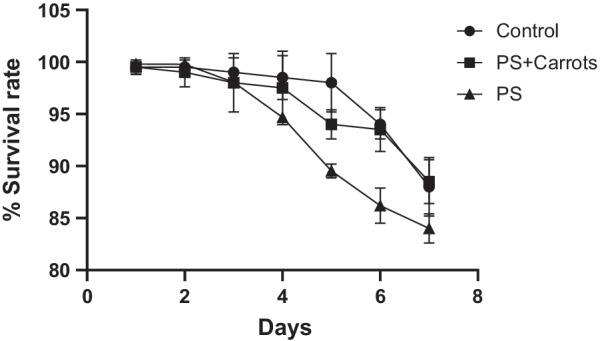
The survival percentage rate of the mealworms from the three groups. The control had the highest survival rate followed by the group of mealworms which were fed on polystyrene and carrots and lastly the group that fed on polystyrene alone had the lowest percentage surviving rate. All values are mean ± SD for N = 2 and P < 0.05 against the control for both tested samples

The number of the larvae that were still surviving from both groups were recorded after seven days and the larvae that developed into pupae were collected and placed in one container containing corn flour and carrots. The percentage survival rate of the mealworms from the three different groups was presented in the form of a line graph as shown in Fig. 5. The control group which had mealworms fed on cornflour and carrots and the second group with mealworms fed on polystyrene and carrots had a survival percentage rate of 90%. However, the third group that had mealworms fed on polystyrene only had a percentage survival rate of 85% which was slightly lower than the percentage survival rates of both group 1 and group 2.

**Fig. 6:**
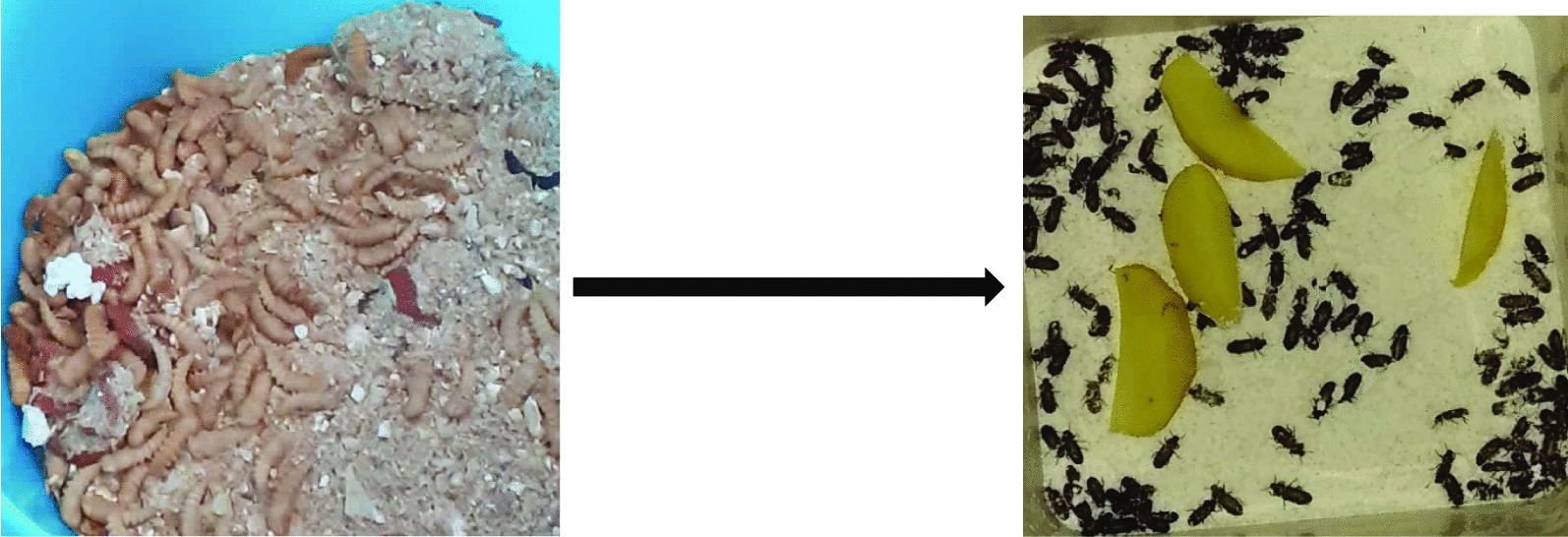
Life cycle of *Tenebrio molitor* completed as pupae collected from the three groups during the experiment finally turned into beetles

Some pupae further developed into adults (darkling beetles). Figure 6 shows the collected pupae and some darkling beetles.

### Isolation of the polystyrene-degrading bacteria from *Tenebrio molitor’s* gut

**Fig. 7:**
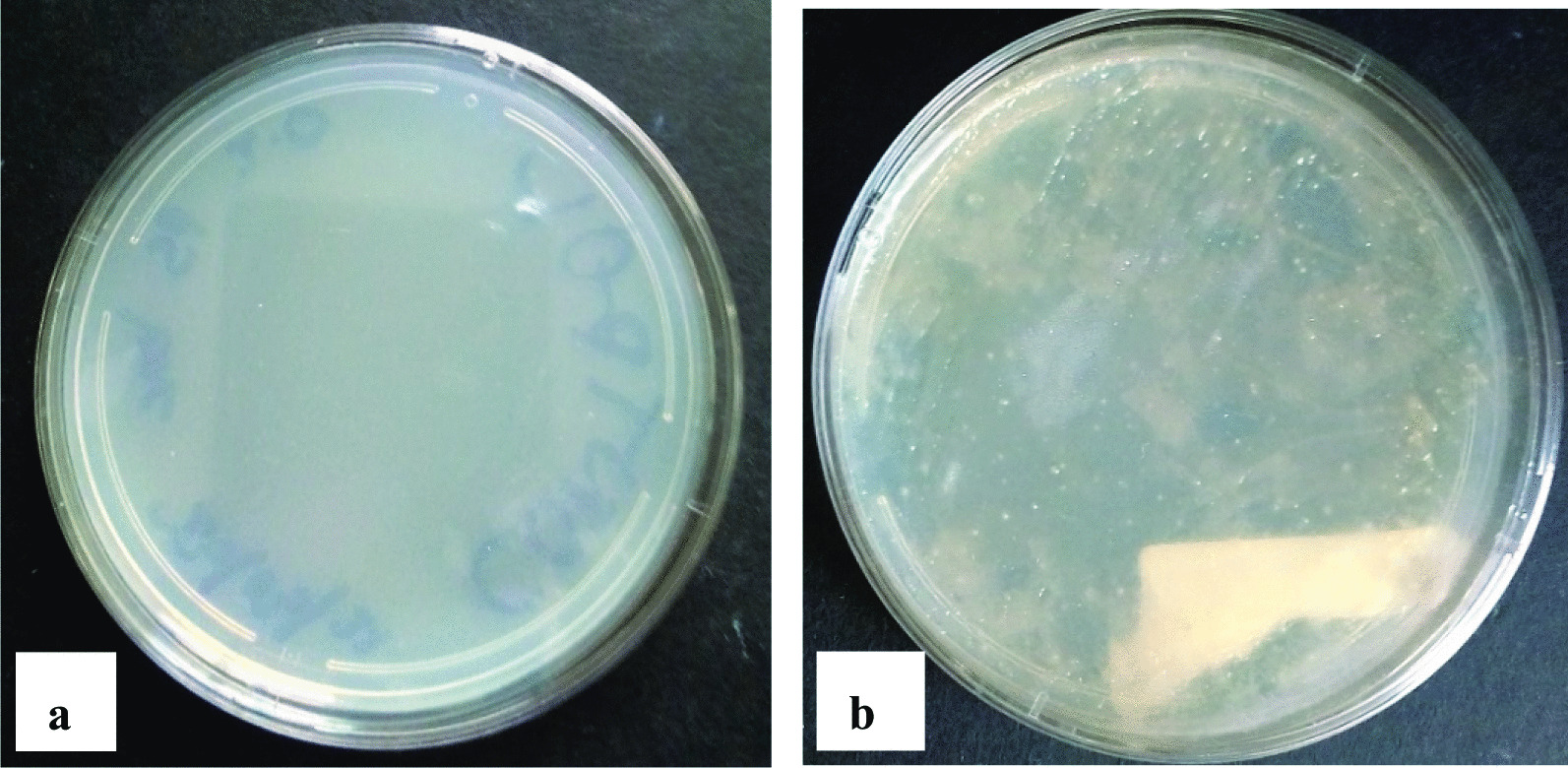
Bacteria isolated from the gut of the larvae of *T. molitor.* **a** control and **b** isolated bacteria

Colonies were observed in the culture plate (Fig. 7) and this shows that the bacteria in the guts of *Tenebrio molitor* was isolated.

**Fig. 8:**
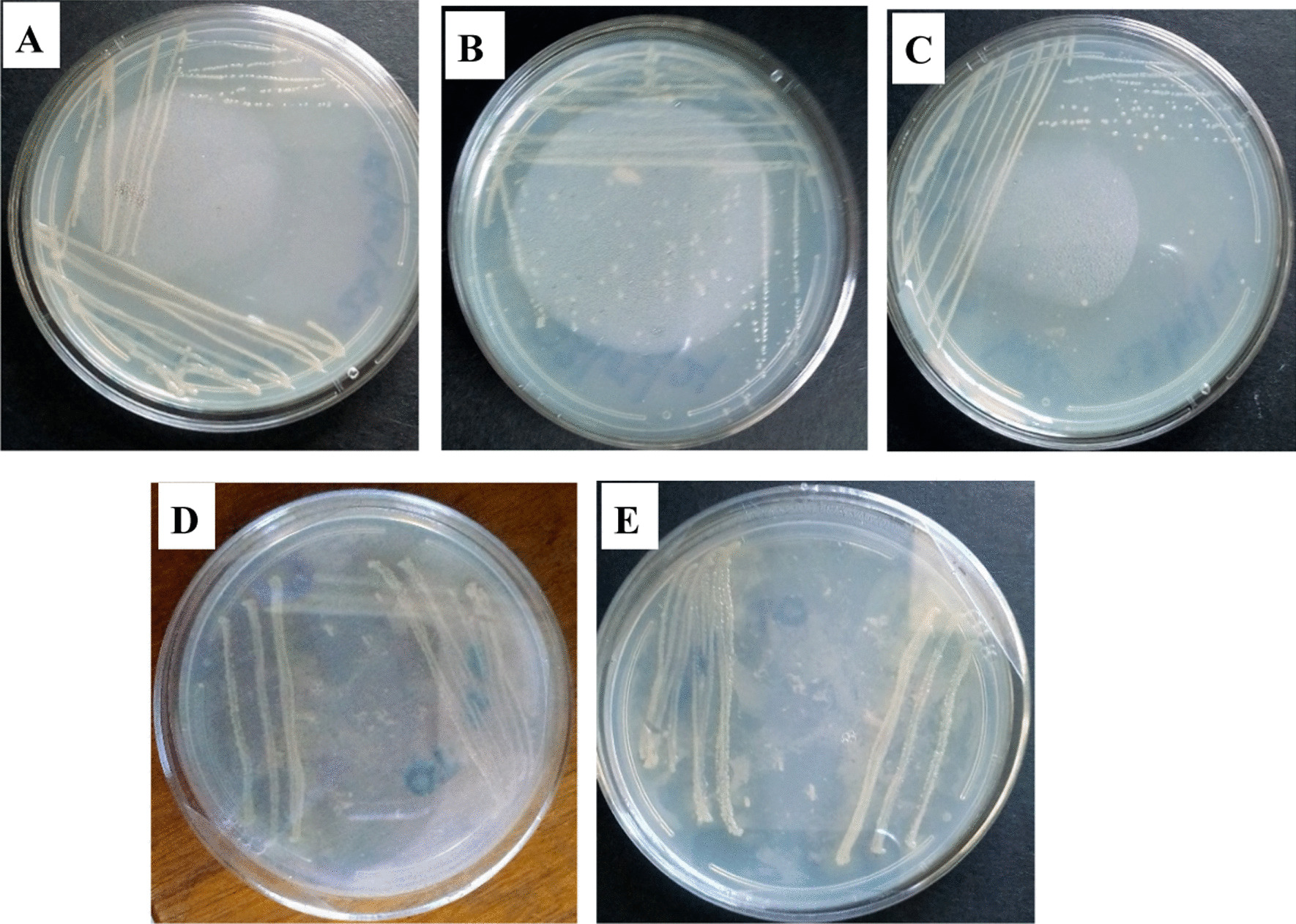
Pure colonies of the *Tenebrio molitor*’s gut microbes obtained after the quadrant streak method

After obtaining the bacteria that was present in the enrichment culture, five colonies were selected and collected as different isolates based on cell morphology which included the shape, colour and size. Two of the colonies were yellow in colour whilst the other three were white. Of the two yellow colonies, one was shiny hence the isolates were recorded as different. The three white colonies appeared different in terms of brightness hence were also recorded as different isolates. The selected colonies were streaked on a fresh polystyrene modified agar plate for sub-culturing that was done repeatedly to get pure colonies A–E as shown in Fig. 8.

**Fig. 9:**
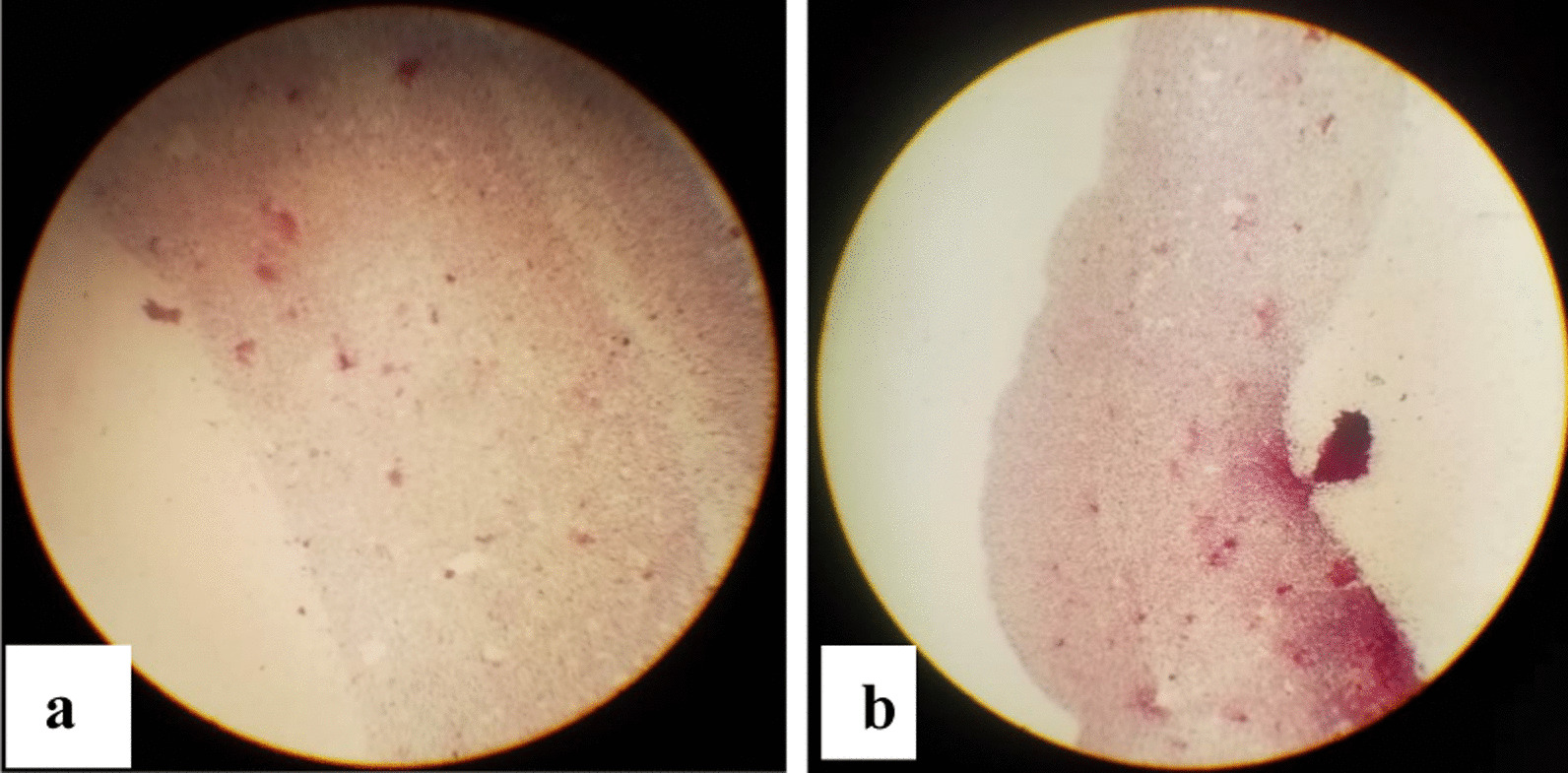
Gram stain of the isolated *Tenebrio molitor’s* gut bacteria. **a** and **b** are only two of the five isolates obtained

The gram stain procedure was carried out and the results show that all the isolates were gram-negative as they all stained pink to red (Fig. 9).

### DNA extraction

**Fig. 10:**
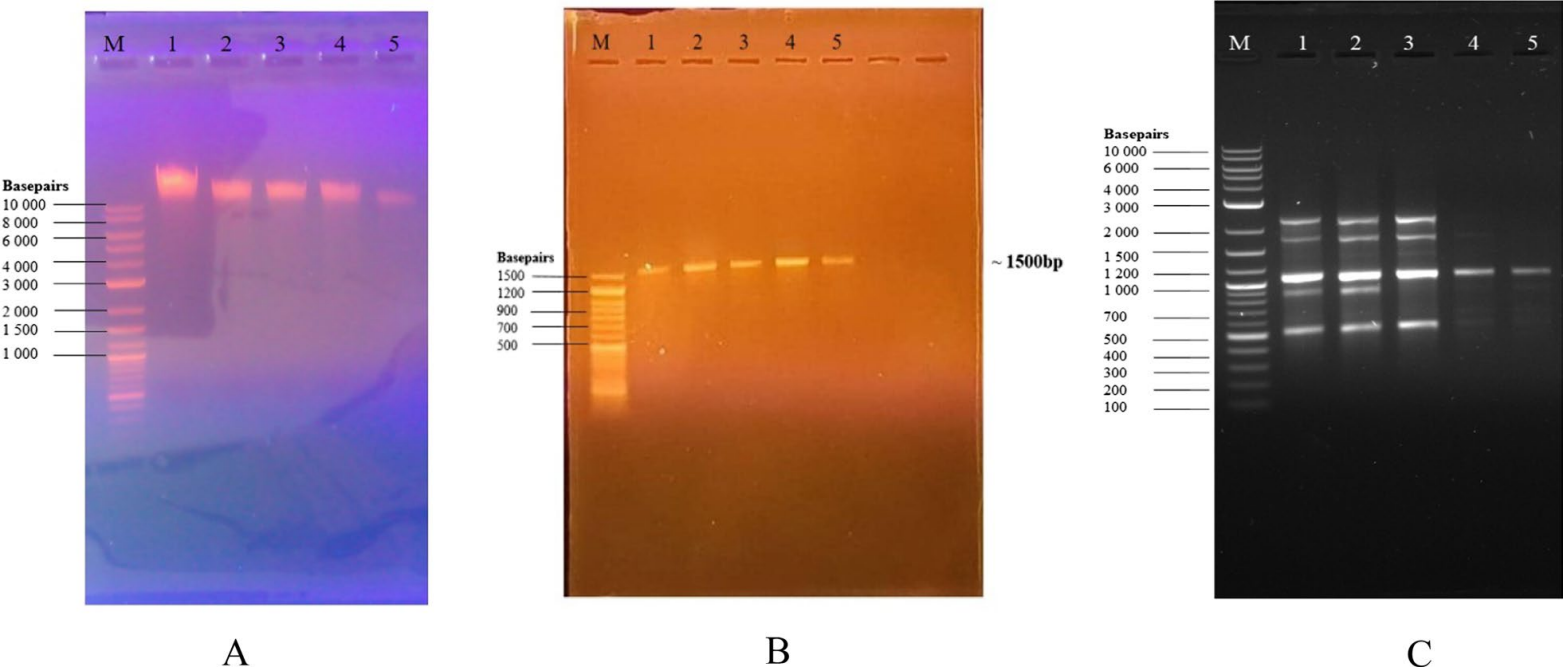
**A** is the agarose gel electrophoresis image of isolated genomic DNA from *Tenebrio molitor*’s gut bacteria ran on 0.8% agarose gel and stained with ethidium bromide. M is 1 kb Plus ladder from New England Biolabs and Numbers 1–5 are the genomic DNA samples isolated from the bacterial samples, **B** is the agarose gel electrophoresis showing the amplification products of the 16S ribosomal RNA genes from the bacterial isolates from the gut of *Tenebrio molitor*. The 16S rRNA gene products were viewed on 1% agarose gel stained with ethidium bromide. M is a 50 bp ladder from Genedirex. Numbers 1–5 are the 16S amplicons and **C** is the agarose gel electrophoresis results after the amplification of DNA from *Tenebrio molitor*’s gut isolates, using the M13 primers. M is a 1 kb plus ladder from NEB. Full-length of the gel is reported in Additional file 2

After confirming the viability of all the five isolates, each of the isolates was cultured in nutrient broth to be used for DNA extraction. Successful isolation of the DNA was confirmed by agarose gel electrophoresis as shown in Fig. 10A The isolates numbers 1–5 are showing clear bands which explains the presence of DNA in all the isolated samples. The high molecular weight DNA obtained confirmed that the DNA isolated is genomic DNA. Each sample of DNA obtained from the 5 isolates was subjected to 16S rRNA gene amplification. The 16S amplicons obtained after amplification with an expected band length of 1500 bp – 1550 bp are shown in Fig. 10B. These results show that the 16S rRNA genes from all the isolates were successfully amplified. The DNA of the 5 isolates from the gut of *Tenebrio molitor* gut were also subjected to M13 RAPD-PCR to investigate the phylogenetic relationship of these isolates. The RAPD-PCR results revealed that isolates numbers 1, 2 and 3 were identical. The results for the amplification of the bacterial DNA using the M13 primers are shown in Fig. 10C.

**Fig. 11:**
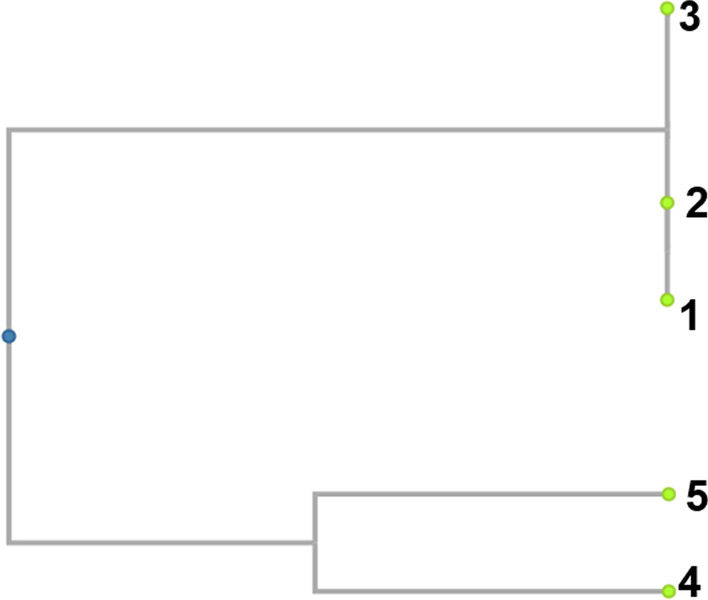
Phylogenetic tree constructed from M13 RAPD-PCR results showing that isolates number 1, 2 and 3 are identical. Uncropped image of the tree is reported in Additional file 2

The phylogenetic tree was then constructed from the M13 RAPD-PCR, using dendroUPGMA. The results, shown in Fig. 11, indicates that isolates 1, 2 and 3 are identical.

Identical isolates 1, 2 and 3 were regarded as one sample and hence this reduced the total number.

of 16S amplicons from 5 to 3.

### Identification of the bacteria based on the nucleotide sequences obtained

**Fig. 12:**
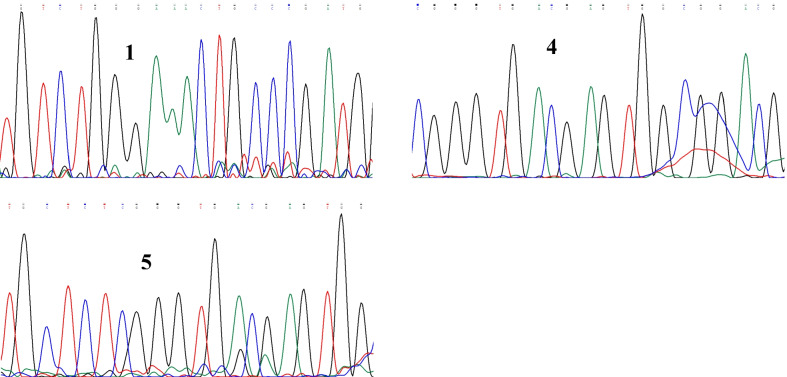
The nucleotide sequences in form of chromatograms for isolates numbers 1, 4 and 5. Each peak represents a single nucleotide in the DNA sequence, and each nucleotide has a different colour, A is green, T is red, C is blue and G is black

The 3 different 16S amplicons were sequenced directly with the 27F primer using the Sanger method. The nucleotide sequences in the form of chromatograms for the three isolates are shown in Fig. 12. The nucleotide sequences in the form of chromatograms for the three isolates were exported to fasta files and the bases were generated for each isolate. Bases obtained for each isolate are reported in Additional file 1. The 16S rRNA sequence generated for isolate 1, 4 and 5 had 517, 539 and 547 bases respectively.

Strain identification by the Basic Local Alignment Search Tool (BLAST) showed that isolate number 1 was *Klebsiella oxytoca* strain ATCC 13182. Since isolate number 1 was identical to isolates numbers 2 and 3, isolates 2 and 3 were also identified to be *Klebsiella oxytoca* strain ATCC 13182. Isolates numbers 4 and 5 were identified as *Klebsiella oxytoca* JCM 1665 and *Klebsiella oxytoca* NBRC 102593, respectively. Table 1 shows percentage similarity of tested strains against representative species in the BLAST search.

**Table 1:** Percentage similarity of tested strains against representative species in the BLAST search

Number of isolate	Representative species	Percentage similarity (BLAST) (%)
1	*Klebsiella oxytoca* ATCC 13182	87.70
2	*Klebsiella oxytoca* ATCC 13182	87.70
3	*Klebsiella oxytoca* ATCC 13182	87.70
4	*Klebsiella oxytoca* JCM 1665	99.77
5	*Klebsiella oxytoca* NBRC 102593	99.77

**Fig. 13:**
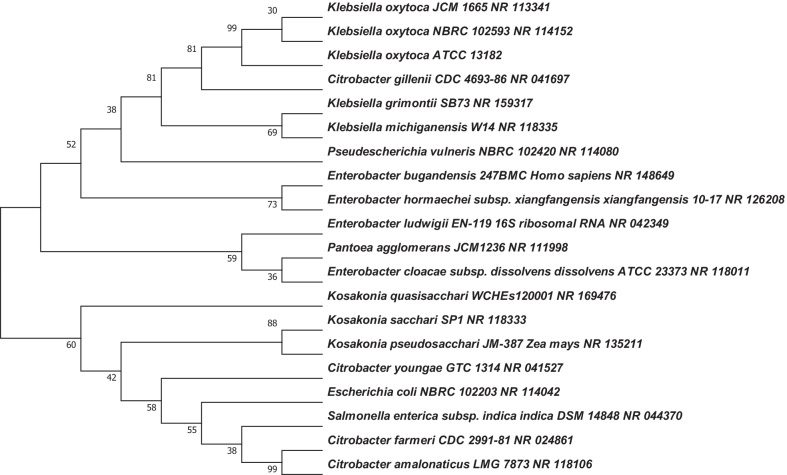
Neighbor-joining tree based on 16S rRNA sequences showing the relationship between isolates number 1, 4 and other closely related species of the genus *Klebsiella*. The evolutionary distances were computed using the Maximum Composite Likelihood method

The maximum Composite Likelihood method for the determination of the evolutionary distances among the isolates gave a phylogenetic tree in Fig. 13. The results show a relationship among isolated bacteria and closely related species of the genus *Klebsiella*. The numbers above the branches are support value obtained from 1000 bootstrap replicates. The external nodes are representing the actual sequences that exist today wheras internal nodes and branches that connect nodes represent the hypothetical ancestors and the lengths of the branches are representing the amount of change that is estimated to have occurred between a pair of nodes.

## Discussion

Polystyrene recycling, as well as cleanup from the environment, is very expensive hence there is a need to look at a biological approach in minimizing environmental issues it poses. This current study focused on cost-effective and affordable research on the digestion of polystyrene by the larvae of *Tenebrio molitor* (yellow mealworms). The results showed that these mealworms survived when they were fed with polystyrene for 7 days, and this indicates that the consumption of the polymer plastic is aided by the gut microbiota. It has also been noted that the survival rate of mealworms fed with polystyrene (85%) was slightly lower than those fed with a normal diet which had 90% (Fig. 5), hence, it is feasible that polystyrene waste can be fed to the mealworms for biodegradation. However, the rate of development of polystyrene fed mealworms to pupae was slower as compared to the control group (Table 1) even though they eventually all developed into pupae and finally into beetles. These obtained results are in line with Morales-Ramos’ who reported that the number of mealworms that survives after 7 days of feeding them varies depending on the type of food available, and that diet affects the development time [8].

The ability of the mealworms to degrade polystyrene is mainly due to the role and activity of gut bacteria [9], thus, *Tenebrio molitor* was dissected and gut collected to isolate the bacteria that may be responsible for the biodegradation. The isolated bacteria were cultured in a medium with polystyrene as the carbon source and then plated on polystyrene modified agar so that those able to biodegrade the polymer could grow. Based on morphology, size and colour, 5 colonies were picked and subcultured until pure colonies were obtained. The five colonies from the *Tenebrio molitor’s* gut were gram stained and all of them stained pink to red, hence, all were gram-negative.

Each of the five isolates was cultured in nutrient broth and DNA extraction of the *Tenebrio molitor’s* gut bacteria was then carried out using bacterial cells that were harvested in their late exponential phase, the phase that involves multiple rounds of DNA synthesis [10]. DNA was successfully isolated from the five isolates from the *Tenebrio molitor*’s gut using the phenol–chloroform DNA extraction method as confirmed by agarose gel electrophoresis which showed high molecular weight DNA (Fig. 10A). The bands show that all the isolated DNA were more than 10,000 bp. Generally, the size of many bacterial genomes ranges from 130 kb to more than 14 Mbp [11]. After isolating the genomic DNA from the five isolates, the near-full length of the 16S rRNA gene was amplified using the universal primer set of 27F/1492R. The primer set that was used was designed to amplify the near-full-length 16S rRNA gene for bacterial identification. A distinct band from each isolate from the *Tenebrio molitor*’s gut was obtained after the amplification of the 16S rRNA genes as shown in Fig. 10B. The PCR yielded products of approximately 1500 bp.

The genetic variation in the five isolates from the gut of *Tenebrio molitor* was identified using random amplified polymorphic DNA (RAPDS), a PCR-based technique. The RAPD-PCR reaction distinguishes nucleotide sequence polymorphisms in a DNA amplification-based assay such that a single species of primer binds to the genomic DNA at two different sites on opposite strands of the DNA template. It was observed that the priming sites were within an amplifiable distance of each other for each DNA template from each of the five isolates since there were discrete DNA fragments that were produced through thermocylic amplification. The DNA products that were produced after RAPD-PCR assay were viewed on agarose gel and the gel image is shown in Fig. 10C. The polymorphisms between isolates resulted from sequence differences in one or both of the primer binding sites, and this is normally shown by the presence or absence of a particular RAPD band as shown in Fig. 10C. Thus, polymorphisms behave as dominant genetic markers. In this study, there were no polymorphisms between DNA from isolates numbers 1, 2 and 3, the DNA from the three isolates had the same number of scores of RAPD bands. This revealed that isolates numbers 1, 2 and 3 were identical. However, for isolates numbers 4 and 5, the polymorphism between them was caused by the sequence differences in both of the primer binding sites, since they did not have the same number of scores of RAPD bands.

The products of RAPD-PCR shown in the gel image were then used to construct a dendrogram or a phylogenetic tree to show the relationship between the isolates from the *T. molitor*’s gut. The dendrogram was constructed based on the score for a band from each of the DNA from the five isolates and the dendrogram is shown in Fig. 11. On the phylogenetic tree, it is clear that isolates numbers 1, 2 and 3 are identical as they are on the same branch. Isolates numbers 4 and 5 are on different branches which indicates that they are different amongst themselves as well as from isolates numbers 1, 2 and 3.

To identify the amplification products, the 16SrRNA gene sequencing was used in this study. The 16S rRNA gene PCR products were first purified to remove excess primers and nucleotides using the QiaQick PCR purification kit (Qiagen) before sequencing. The 16S rRNA amplicons were then sequenced with the Seqstudio genetic analyser using the Sanger method. The electropherograms for the fragments generated for isolates numbers 1, 4 and 5 are shown in Fig. 12. The obtained sequences were identified by the BLAST tool to be *Klebsiella oxytoca* ATCC 13182 for isolate number 1, *Klebsiella oxytoca* JCM 16655 for isolate number 5 and *Klebsiella oxytoca* NBRC 102593 for isolate number 4 after comparison with sequences in the GeneBank in NCBI database. The percentage identity of isolate number 1 with *Klebsiella oxytoca* ATCC 13182 was 87.70%, that of isolate number 4 with *Klebsiella oxytoca* NBRC 102593 was 97.92% and that of isolate number 5 with *Klebsiella oxytoca* JCM 16655 was 99.78%.

*Klebsiella oxytoca* is a Gram-negative, rod-shaped bacterium that is closely related to *K. pneumonia*. However, it differs from *K. pneumonia* in that it is indole-positive [12], as confirmed by the oxidase test which was carried out on the isolates from the mealwoms gut. It is reasonable to assume that polystyrene-degrading bacteria have features present in broad families of aerobic or facultative bacteria and can secrete extracellular oxidative enzymes that are responsible for the breaking down of polystyrene polymer chains.

Obtained sequences were used to estimate the relationships among taxa or sequences to construct a phylogenetic tree as shown in Fig. 13. The phylogenetic tree shows the relationship of the three *Klebsiella oxytoca* strains that were isolated from the *T.molitor* gut with closely related microorganisms. The bootstrap percentages reveals the reliability of the cluster descending from every node such that the higher the number, the more reliable would be the estimate of the taxa that descend from a particular node. From the phylogenetic tree in Fig. 13, the nodes that had higher percentages had the following taxa that descended from them and these include *Citrobacter gillenni*, *Enterobacter hormaechei subp*, *Kosakonia psuedosacchari* and *Citrobacter amalonaticus*. These taxa are the ones reliable in estimating their relationship with *Klebsiella oxytoca* ATCC 13182. The estimated taxa could be true as there are previous studies that observed them in the biodegradation of polystyrene. *Enterobacter hormaechei* has previously been isolated from the *T.molitor* [13].

Thus, in this study, the bacterial community found in mealworms was primarily composed of the Proteobacteria phylum. Mostly four dominant phyla namely Proteobacteria, Firmicutes, Tenericutes and Actinobacteria have been obtained in other researches on mealworms fed with polystyrene [13]. Differences in results obtained from this study and other researchers show that bacterial community composition could be completely different among samples within the same host as observed by Jung who tested nine individual mealworms and noted that the bacterial communities were not identical across all individuals [14]. This observation confirmed the theory of Cariveau who proposed that bacterial communities can be different between individuals growing in the same environment [15]. The results might suggest that mealworms from different areas have a part of their microbiome in common. In another study, differences between mealworms from diverse locations were observed and it was revealed that *T. molitor* from different regions presents different microorganisms responsible for the biodegradation of polystyrene [16; 17].

## Conclusions

The bacterial community found in mealworms in this study was primarily composed of the Proteobacteria phylum. The 16S ribosomal RNA gene sequencing revealed that all of the five *T.molitor* larval gut isolates were of the *Klebsiella oxytoca* species but were of different strains. The isolated bacteria were *Klebsiella oxytoca* ATCC 13182, *Klebsiella oxytoca* NBRC 102593 and *Klebsiella oxytoca* JCM 16655. The obtained results suggest that the gut of *T. molitor* is a very good source for microorganisms capable of biodegrading polystyrene. However, further work is needed to determine the capacity of the isolates by isolating the enzymes which may be responsible for plastic degradation and investigating their kinetics.

## Methods

### Mealworms

The mealworms (larvae of *Tenebrio molitor*) were directly purchased at their growth age of approximately 5–7 instar from a local company named National Hatch Web, which is located in Harare in Zimbabwe. Based on morphology and colouration, the mealworms were identified as *Tenebrio molitor Linnaeus.* Upon their arrival, the mealworms were fed with bran for two days before polystyrene degradation tests were carried out.

### Extended polystyrene (EPS) feedstocks

The expanded polystyrene (EPS) to be tested for biodegradation were collected as recycle waste. The mealworms were fed with EPS as feedstocks. These EPS feedstocks had similar densities and similar molecular weights.

### Polystyrene biodegradation capability

The mealworms were cultivated in polypropylene (PP) storage containers in the laboratory and the set-up and methods that were used were the ones previously reported [17]. The mealworms were divided and allocated into three groups, with different feeding conditions, with 100 mealworms in each group. The mealworms were reared for 1 week (7 days). The temperature was maintained at 25 ºC and humidity was maintained at 75–80% in each container. The first group was the control hence mealworms were fed with normal feed which is cornflour and carrots, the second group was fed with 1.5 g of expanded polystyrene and carrots and the third group was fed with 1.5 g of expanded polystyrene foam only. In all three groups, dead mealworms were removed whenever observed. The number of surviving larvae and pupae were recorded and polystyrene mass loss was monitored and calculated.

### Characterization of feedstock and frass

Biodegradation and depolymerization of ingested polystyrene were assessed by visual observation of polystyrene as feedstock and the frass egested. The visual observation was done to evaluate the macroscopic changes such as roughening or scabrous and the creation of holes and cracks, on the surface structure of the polymer. Visual changes were used as the first indication of *Tenebrio molitor* larvae attack on the polystyrene polymer [18]. The biodegradation was also determined by measuring the mass loss of the polystyrene feedstock.

### Isolation of polystyrene-degrading bacteria

The gut cell suspension was prepared from a total number of 50 mealworms which were fed with polystyrene as a sole source of carbon in the diet. The mealworms were sterilized by immersing them in 75% ethanol for 1 min followed by rinsing with sterile 0.85% saline water. The mealworms were then dissected to remove the entire gut from each larva, using surgical blades and forceps. The guts were placed in a sterile petri dish and the midguts were drawn out and pooled in a 10 ml centrifuge tube containing 5 ml of saline water. The collected samples were then homogenized for 10 min using a Dounce homogenizer. The gut tissues were removed using a pipette. The gut cell suspension was stored at 4 °C for later use as the inoculum of the polystyrene-degrading bacterial enrichment.

### Preparation of the polystyrene emulsion and agar

The polystyrene emulsion for microbial degradation was prepared by dissolving EPS feedstocks in dichloromethane solvent at 3%. The solution was then transferred to an amber bottle containing the same volume of liquid carbon-free medium (LCFBM) and was stored at 4 ºC for two days. After two days the polystyrene emulsion was placed in a fume hood overnight to volatilize the dichloromethane solvent. The polystyrene agar was prepared by transferring 50 ml of polystyrene emulsion solution to an Erlenmeyer flask containing 50 ml of liquid carbon-free medium with 1.5 g of agar powder and was autoclaved at 121 ºC for 15 min.

### Isolation and identification of microorganisms

The collected gut cell suspension was used as the inoculum of polystyrene-degrading bacterial culture enrichment. The gut cell suspension was inoculated into an Erlenmeyer flask containing polystyrene emulsion as the sole carbon source, and liquid carbon-free basal medium (LCFBM). The flask that served as the control contained polystyrene emulsion and LCFBM only. The flasks were then incubated on a rotary shaker at 120 rpm and an ambient temperature of 30 °C for 28 days. After 28 days of the incubation period, the enrichment was spread on a plate with modified polystyrene agar and then incubated for 24 h at ambient temperature to enumerate the number of bacteria present in the enrichment culture. Colonies from the plate were then selected based on cell morphology (shape, colour, and size) and streaked on fresh polystyrene agar plates for sub-culturing. The quadrant streak method was employed to obtain pure colonies. The pure colonies of the isolates were Gram-stained and viewed under a light microscope for bacteria characterization.

### Molecular identification

After obtaining pure colonies, each of the isolates was inoculated in 100 ml nutrient broth in 250 ml conical flasks and incubated in a rotary shaker at 30 ºC at 120 rpm. The growth of the isolates was monitored by measuring their optical density at 600 nm, after every hour. Cell suspension for DNA extraction was collected from each isolate in its late exponential phase.

### DNA extraction and amplification

DNA extraction was carried out through precipitation and centrifugation using phenol–chloroform DNA extracting protocol. DNA was visualised after electrophoresis at 120 V and 400 mA for 30 min on agarose gel (0.8% (w/v) in TAE buffer, stained with Ethidium Bromide solution). The concentration of DNA was determined by nanodrop spectrophotometer. The supernatant containing the extracted DNA was used to amplify 16S ribosomal DNA segments through PCR using 16S universal primers 27 F (5′-AGAGTTTGATCCTGGCTCAG-3′) and 1492R (5′-TACGGYTACCTTGTTACGACTT-3′). The PCR of the 16S rRNA gene with universal primers contained 5 µl of 5X One *Taq* reaction buffer, 0.5 µl of 10 mM dNTPs, 0.5 µl of 10 µM forward primer, 0.5 µl of 10 µM reverse primer, 1 µl of template DNA, 0.125 µl of *Taq* Polymerase, 1 µl of 25 mM MgCl_2_ and was brought up to a final volume of 25 µl with ultra-pure water. The reactions were performed in a thermocycler under the following conditions: 5 min at 95 ºC, followed by 35 cycles of 45 s at 95 ºC, 40 s at 56 ºC, 2 min at 72 ºC and a final extension step at 72 ºC for 5 min. The expected band length for the PCR product was 1500 bp. The PCR products were visualised after electrophoresis at 150 V and 400 mA for 30 min on agarose gel (0.8% (w/v) in TAE buffer, stained with Ethidium Bromide solution). The PCR products were directly sequenced with using the Sanger sequencer. The 16S rRNA sequences obtained were viewed using Chromas 2.6.6 software. The obtained sequences were subjected to BLAST search in NCBI database for phylogenetic relationship.

### M13 RAPD-PCR

The M13 RAPD-PCR was carried out to investigate phylogenetic relationship among isolates. The RAPD-PCR contained 12.5 µl of mastermix, 1 µl of the M13 forward primer, 1 µl of the reverse primer, 1 µl of DNA and 9.5 µl of ultra-pure water. The reactions were performed in a thermocycler under the following conditions: 1 min at 95 ºC, followed by 40 cycles of 1 min at 95 ºC, 30 s at 38 ºC, 2 min at 72 ºC and a final extension step at 72 ºC for 10 min.

### Phylogenetic tree construction

The 16S rRNA sequences obtained were viewed using Chromas 2.6.6 software. The Basic Local Alignment Search Tool (BLAST) was used to analyse the obtained sequence with organisms in the Gen Bank database for strain identification and construction of phylogenetic tree analysis. The phylogenetic tree and molecular evolutionary analyses were constructed using MEGAX software with the Neighbour Joining algorithm. The percentage of replicate trees in which the associated taxa clustered together in the bootstrap test (1000 replicates) was shown next to the branches. The evolutionary distances were computed using the Maximum Composite Likelihood method.

## Supplementary Information

**Additional file 1.** Bases for isolates 1, 4 and 5.

**Additional file 2.** Phylogenetic tree constructed from M13 RAPD-PCR results showing that isolates 1, 2 and 3 are identical.

## Data Availability

All data generated or analysed during this study are included in this manuscript [and its supplementary information files].
