# Supplementary material for: Tenebrio molitor: possible source of polystyrene-degrading bacteria
Source: BMC Biotechnol. 2022 Jan 4;22:2. doi: 10.1186/s12896-021-00733-3 (PMC8728996; doi:10.1186/s12896-021-00733-3)
Supplement: Supplementary file 1 — Additional file 1. Bases for isolates 1, 4 and 5. [file 12896_2021_733_MOESM1_ESM.docx]

**Additional file 1.**

The 3 different 16S amplicons were sequenced directly with the 27F primer using the Sanger method. The nucleotide sequences in the form of chromatograms for the three isolates were exported to fasta files and the bases were generated for each isolate.

1. The 16S rRNA sequence generated for isolate 1. The sequence had 517 bases.

TCGTGGGGGGGCCGWGGCCCTTAMACATGCAAGCTCKAAACGGTARAAAAAGGGGGGGAA

GSTCTCGGGTGACGAGTGTTARRTAAAGGARAAGWAAGTCTGGGAAACTGCCCGATGGAR

GGGGATAACTACTGGAAATTTTTARWTAAKACCGCATACGTCGCAAGACCAAAGAKGGGG

ACCTTMRGGCCTCTTGCCATCGGATGTGCCCRATGGGATTAGCTTGTAGTGAGGTAACGG

CTCACCTAGCGACGATCCCTAGCTGGTCTGAGAGGATGACCARCCACACTGGAACTGAGA

CACGGTCCARACTCCTAGKGGAGGGCAGCRKTGGGGAATATTGCACAATGGGGCGRAAGC

CTTGATGCAKCCATTGCCGCCGTGTATGAAGAAGGCCTTCRGGGTTTGWAAARTTWCTTT

TCASCCGGKGAGGAAAAGGGAATTGMAGGTTTAATAACCCTTTTTTCCTTTRACGTTTTA

CCCCACCARAAAAAAGSCWCCCKGGTTAAAYTTTYCC

1. The 16S rRNA sequence generated for isolate 4. The sequence had 539 bases.

ACKGGGCGGCARGCCGTACACATGCAAGTCGAACGGTAGCATARARAGMTTGCTCTCGGG

TGACGAGTGGCGGACGGGTGAGTAATGTCTGGGAAACTGCCCGATGGAGGGGGATAACTA

CTGGAAACGGTAGCTAATACCGCATAACGTCGCAAGACCAAAGAGGGGGACCTTMGGGCC

TCTTGCCATCGGATGTGCCCAGATGGGATTAGCTTGTAGGTGAGGTAACGGCTCACCTAG

GCGACGATCCCTAGCTGGTCTGAGAGGATGACCAGCCACACTGGAACTGAGACACGGTCC

AGACTCCTACGGGAGGCAGCAGTGGGGAATATTGCACAATGGGCGCAAGCCTGATGCAGC

CATGCCGCGTGTATGAAGAAGGCCTTCGGGTTGTAAAGTACTTTCAGCGGGGAGGAAGGG

AGTGAGGTTAATAACCTTATTCATTGACGTTACCCGCAGAAGAAGCACCGGCTAACTCCG

TGCCAGCAGCCGCGGTATTACGGAGGGTGCAGCGTTATCGGAATTACTGGGGCGTAAAG

1. The 16S rRNA sequence generated for isolate 5. The sequence had 547 bases.

CAAYGGGTKKGGGGGGGYKGGCCGTACACATGSCWWGGTCGMACGGTAGTAGTRAAGAAG

CTTGCTCTCGGGTGACGAGTGGCGGACGGGTGAGTAATGTCTGGGAAACTGCCCGATGGA

GGGGGATAACTACTGGAAACGGTAGCTAATACCGCATAACGTCGCAAGACCAAAGAGGGG

GACCTTMGGGCCTCTTGCCATCGGATGTGCCCAGATGGGATTAGCTTGTAGGTGAGGTAA

CGGCTCACCTAGGCGACGATCCCTAGCTGGTCTGAGAGGATGACCAGCCACACTGGAACT

GAGACACGGTCCAGACTCCTACGGGAGGCAGCAGTGGGGAATATTGCACAATGGGCGCAA

GCCTGATGCAGCCATGCCGCGTGTATGAAGAAGGCCTTCGGGTTGTAAAGTACTTTCAGC

GGGGAGGAAGGGAGTGAGGTTAATAACCTTATTCATTGACGTTACCCGCAGAAGAAGCAC

CGGCTAACTCCGTGCCAGCAGCCGCGGTAATACGGAGGGGGTGCAAAGCCGTTTAAATCC

GGGGAAT
