# Supplementary material for: Tenebrio molitor: possible source of polystyrene-degrading bacteria
Source: BMC Biotechnol. 2022 Jan 4;22:2. doi: 10.1186/s12896-021-00733-3 (PMC8728996; doi:10.1186/s12896-021-00733-3)
Supplement: Supplementary file 2 — Additional file 2. Phylogenetic tree constructed from M13 RAPD-PCR results showing that isolates 1, 2 and 3 are identical. [file 12896_2021_733_MOESM2_ESM.docx]

**Additional file 2**

1. Original full-length gel and blot images.

Agarose gel electrophoresis was used to separate and identify DNA and amplification products of the 16S ribosomal RNA genes.

1. Isolated genomic DNA from *Tenebrio molitor*’s gut bacteria


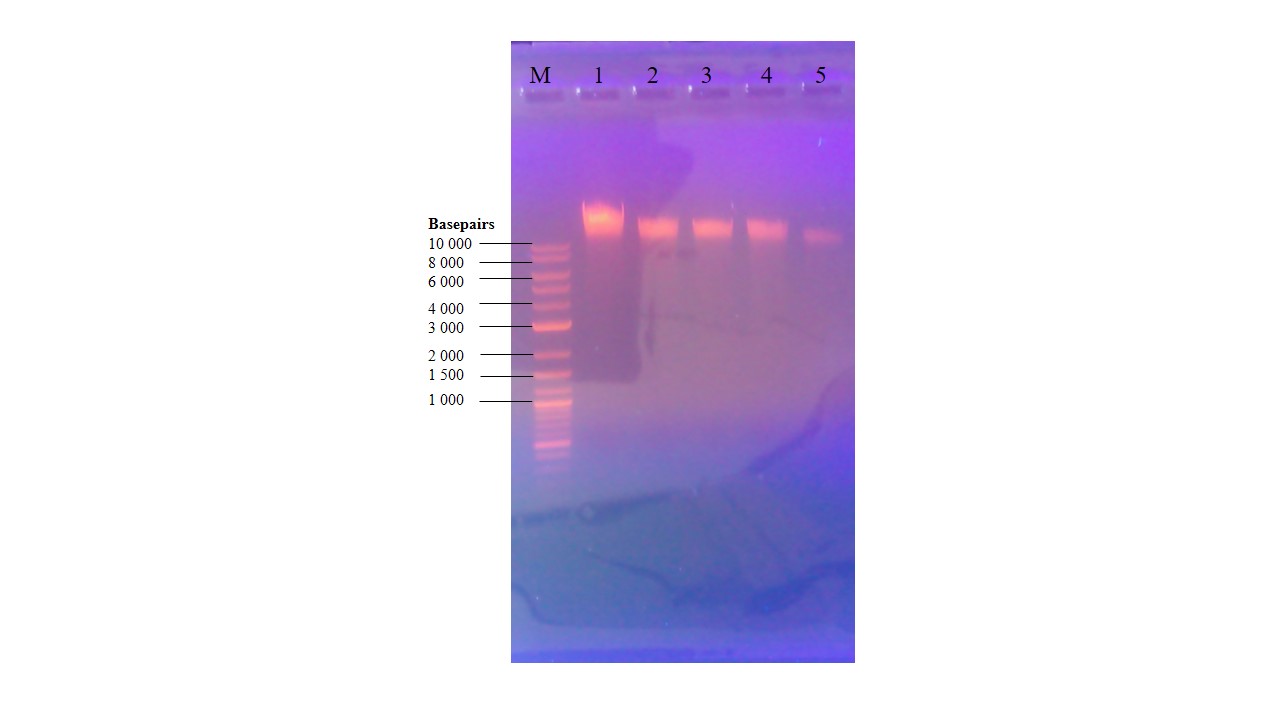


**Figure 10:** Agarose gel electrophoresis of isolated genomic DNA from *Tenebrio molitor*’s gut bacteria ran on 0.8 % agarose gel and stained with ethidium bromide. M is 1kb Plus ladder from New England Biolabs and Numbers 1-5 are the isolated bacterial samples.

1. Amplification products of the 16S ribosomal RNA genes from the bacterial isolates


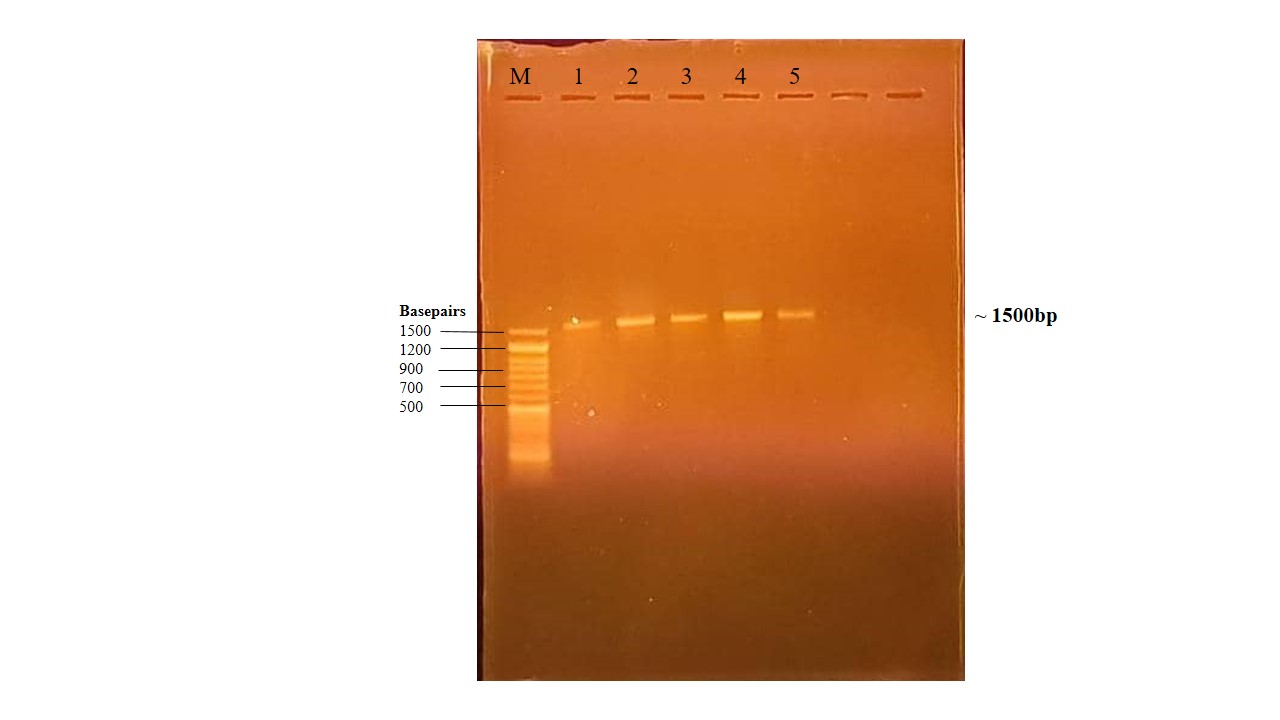


**Figure 11:**  Agarose gel electrophoresis showing the amplification products of the 16S ribosomal RNA genes from the bacterial isolates from the gut of *Tenebrio molitor*. The 16S rRNA gene products were viewed on 1 % agarose gel stained with ethidium bromide. M is a 50 bp ladder from Genedirex. Numbers 1 -5 are the 16S amplicons.

1. After amplification using M13 primers


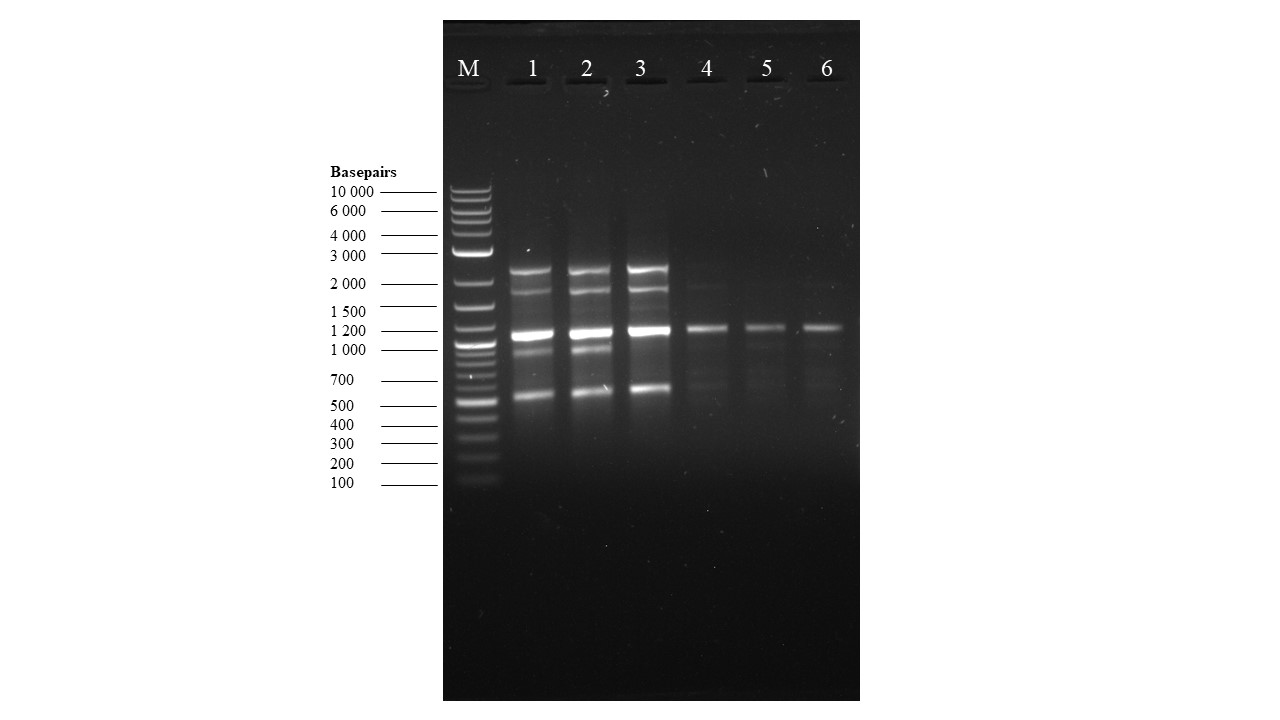


**Figure 12:** Agarose gel electrophoresis results after the amplification of DNA from *Tenebrio molitor*’s gut isolates, using the M13 primers. M is a 1kb plus ladder from NEB.

1. Original, uncropped phylogenetic tree images

The Basic Local Alignment Search Tool (BLAST) was used to analyze the obtained sequence with organisms in the Gen Bank database for strain identification and construction of phylogenetic tree analysis.

1. Phylogenetic tree constructed from M13 RAPD-PCR


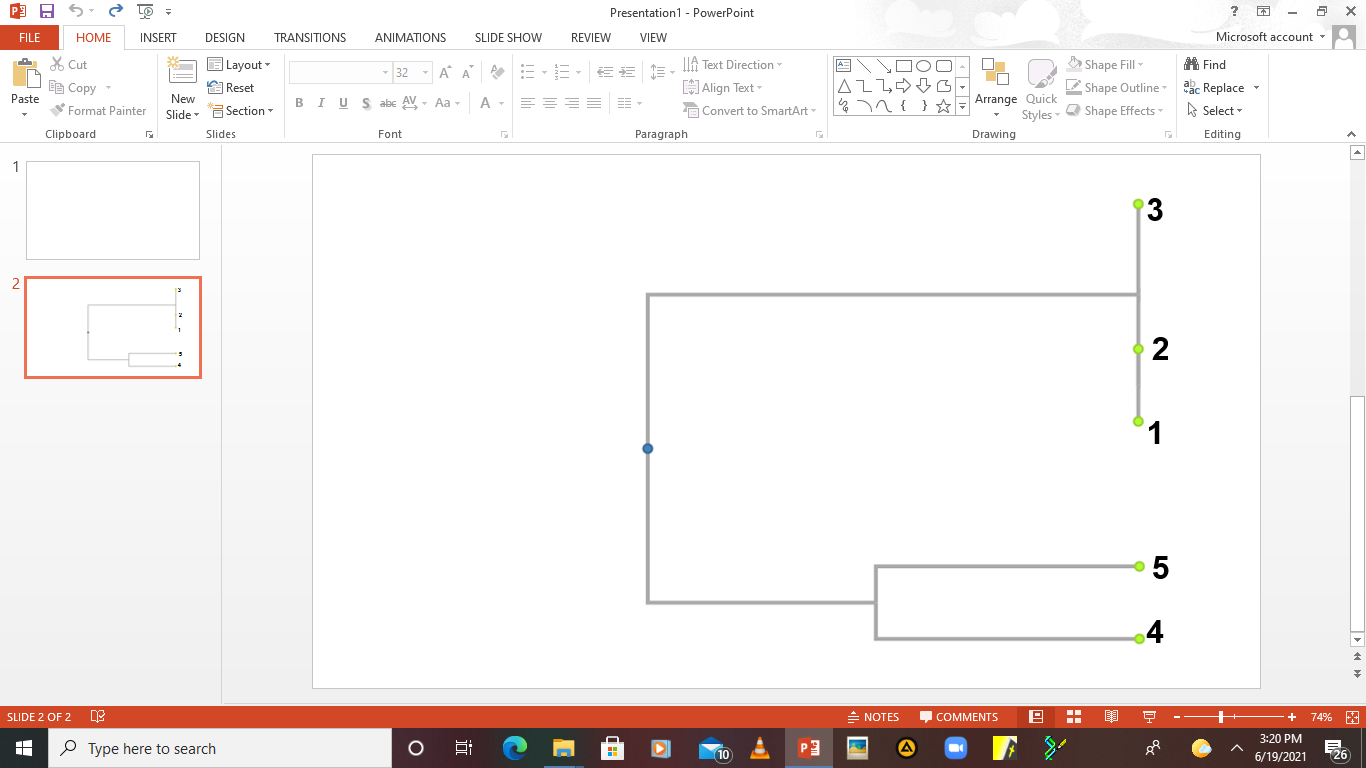


**Figure 13:** Phylogenetic tree constructed from M13 RAPD-PCR results showing that isolates number 1, 2 and 3 are identical.

1. Phylogenetic tree constructed after the 16S rRNA gene nucleotide sequencing


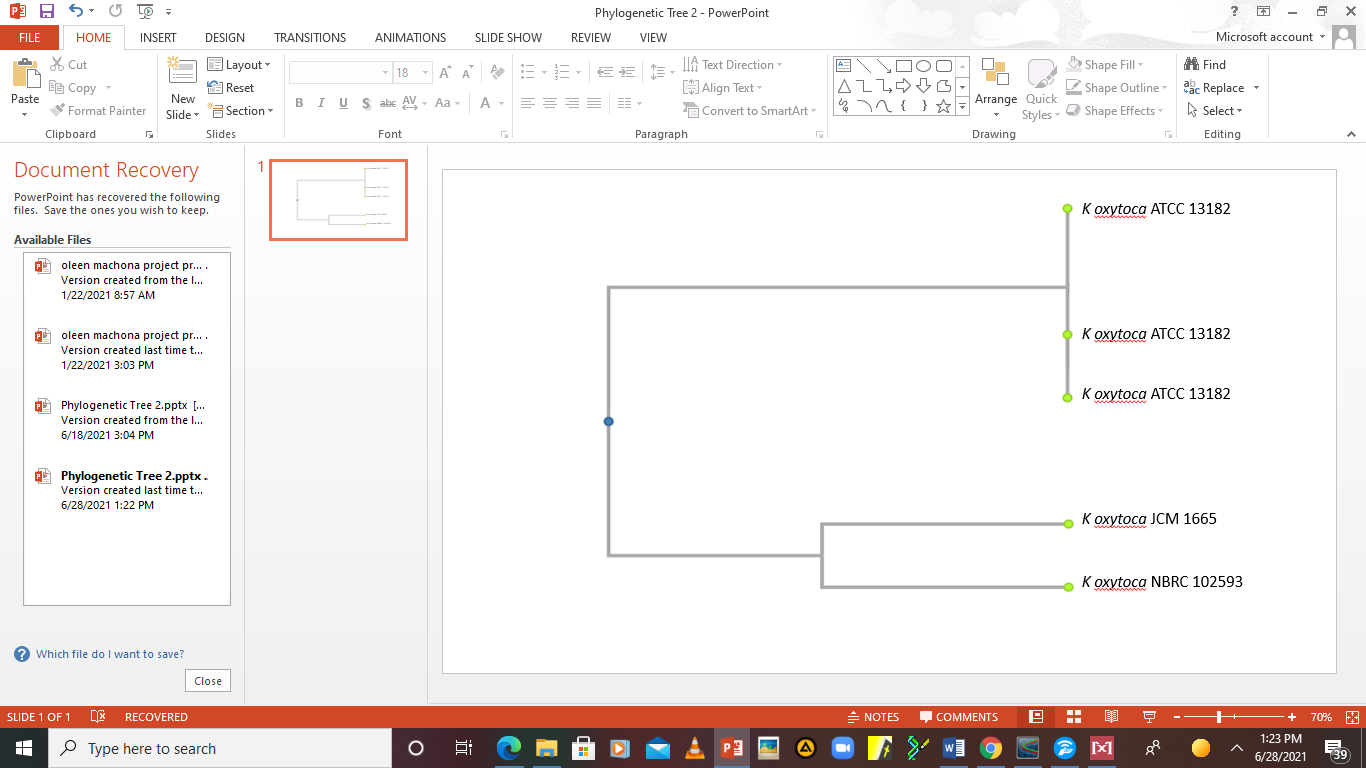


**Figure 15:** Phylogenetic tree constructed after the 16S rRNA gene nucleotide sequencing. Isolates numbers 1, 2 and 3 were identified to be *Klebsiella oxytoca* ATCC 13182, isolate number 4 was identified to be *Klebsiella oxytoca* NBRC 102593 and isolate number 5 was identified to be *Klebsiella oxytoca* JCM 1665.
